# Effects of Different Melting Technologies on the Purity of Superalloy GH4738

**DOI:** 10.3390/ma11101838

**Published:** 2018-09-27

**Authors:** Zhengyang Chen, Shufeng Yang, Jinglong Qu, Jingshe Li, Anping Dong, Yu Gu

**Affiliations:** 1 School of Metallurgical and Ecological Engineering, University of Science and Technology Beijing, Beijing 100083, China; chenzhengyang@xs.ustb.edu.cn (Z.C.); lijingshe@ustb.edu.cn (J.L.); 2 Beijing Key Laboratory of Special Melting and Preparation of High-End Metal Materials, Beijing 100083, China; 3 Beijing Central Iron and Steel Research Institute, Beijing 100081, China; 13810256459@139.com (J.Q.); 18810654973@163.com (Y.G.); 4 School of Materials Science and Engineering, Shanghai Jiao Tong University, Shanghai 200240, China

**Keywords:** superalloy, melting technology, purity, inclusions, mechanical properties, white spot

## Abstract

The choice of melting technique is crucial for controlling the purity of a superalloy, which is especially important because purity has come to limit progress in the superalloy field. In this study, double- and triple-melting techniques were used to refine the GH4738 superalloy. Elemental analyses, inductively coupled plasma-atomic emission spectroscopy, X-ray diffraction analysis, scanning electron microscopy with energy-dispersive spectroscopy, high-temperature cupping machine, high-temperature fatigue testing machine, and Image-Pro Plus software were used to analyze and compare the contents of specific elements, the types and sizes of inclusions, the mechanical properties, and the probabilities of white spot formation using the two melting techniques. The effects of the different melting processes on the purity of the superalloy were systematically studied. In terms of controlling the presence of impurities, the triple-melting process resulted in lower levels of harmful N, S, and O impurities in the superalloy, the triple-melted superalloy also contained fewer types of inclusion of smaller sizes and in smaller amounts than the double-melted alloy. Triple melting also promotes tensile strength and fatigue life, and minimizes the probability of forming defects in the superalloy.

## 1. Introduction

Superalloys, owing to their excellent high-temperature strengths and good resistance to oxidation, fatigue, and creep deformation [1,2,3], have emerged as materials for components that operate at high temperatures in the fields of aerospace and aviation engineering. These include turbine disks and blades, and combustion chambers in aerospace engines; thus, these alloys enjoy reputations as “cornerstones for advanced engines” [4,5,6].

As aerospace and aviation engines become more reliable, develop higher thrust-to-weight ratios, and become larger in size, the presence of various types of inclusions in superalloys, and high percentages of harmful elements that reduce the purities of these materials, pose threats to their operating safety and stability [7,8,9,10]. Wang et al. [11] found that the formation of carbon nitride inclusions during the cooling and crystallization of a superalloy lowers its plasticity and service lifetime. As shown in previous studies [12,13], sulfur has a strong tendency to segregate at grain boundaries in nickel-based superalloys and readily forms low-melting-point eutectic products that are brittle at high temperatures, seriously affecting the plasticities of these alloys for thermal processing. Song et al. [14] examined the Inconel718 alloy and found that the instantaneous tensile strength, plasticity, and creep resistance of the alloy at both room temperature and elevated temperatures decreased with increasing sulfur content, while creep-rupture life and creep-rupture ductility both dropped sharply. On the basis of the above discussion, strict control of superalloy purity is of particular importance in order to meet the increasing material-performance demands of aerospace engineering.

Presently, little enhancement in superalloy purity can be achieved by changing the chemical composition of the superalloy, adjusting the forging process, or improving the thermal processing technique [15,16,17]. Adjusting the superalloy melting process, on the other hand, can lead to significantly improved purity. Degawa et al. [18] used a CaO crucible for the melting and refining of IN738 and MarM247 alloys by vacuum induction melting (VIM), and determined the N, O, and S contents of each to be less than 10 ppm in the produced ingots, representing inappreciably improved metallurgical quality. Instead of using direct current, Wang et al. [19] obtained high-purity ingots by applying low-frequency alternating current and adding a transverse magnetic field during electroslag remelting (ESR). Shevchenko et al. [20] showed that the time-variation and asymmetric distribution of the arc during vacuum arc remelting (VAR) hinder slag discharge by the molten pool, which affects the purity of the ingots. As such, appropriate control of the arc may effectively improve ingot quality. 

To summarize, researchers have substantially improved superalloy purity by adjusting the conditions of the melting technique [18,19,20]. However, there have been few reports on the influence of the overall melting process on superalloy purity, especially regarding the influence of the melting technique itself. Therefore, it is difficult to precisely determine the melting technique best suited for the preparation of a high-purity superalloy, which limits further improvements in superalloy performance. To overcome this problem, the effects of different melting techniques on superalloy purity should be studied. In this study, double (VIM + VAR) and triple (VIM + ESR + VAR) melting techniques were used to refine the GH4738 superalloy. The contents of specific elements at different melting stages and mechanical properties in the superalloy were measured. The types, amounts, and sizes of inclusions were observed and recorded to analyze and compare the effects of the two different melting processes on the purity and properties of the superalloy. This research serves as a guide for the selection of appropriate melting techniques for improving superalloy purity; in this sense, it also provides a theoretical foundation.

## 2. Experimental

### 2.1. Melting

In this experiment, electrolytic Ni plates, high-purity Cr and Mo, revert GH4738, and alloys of other elements were used as raw materials. The GH4738 superalloy was melted by VIM (Consarc, Rancocas, NJ, USA). The vacuum was maintained at around 2 Pa during the entire melting process. Argon was injected prior to the addition of trace elements to prevent volatilization and its effect on alloy composition accuracy. Two alloy ingots of diameters of 460 mm (#1) and 360 mm (#2) were cast at 1400 °C. Their main chemical compositions are listed in Table 1.

Risers were removed from the two ingots and each ingot surface was polished to prevent secondary contamination during ingot melting by refractory and surface oxide coatings. Ingot #2 was then re-melted in a protective gas ESR (ALD, Hanau, Germany) furnace with CaF_2_, Al_2_O_3_, CaO, MgO, and TiO_2_ as the slag components. Slag-melting of the ingot required about 80 min. The melt rate in the steady melting stage was maintained at 3–4 kg/min. Argon was injected at a flow rate of 100 L/min as the protective gas during the entire melting and refining process. A Ф 460-mm (#2) alloy ingot was obtained as the product.

**Figure 1:**
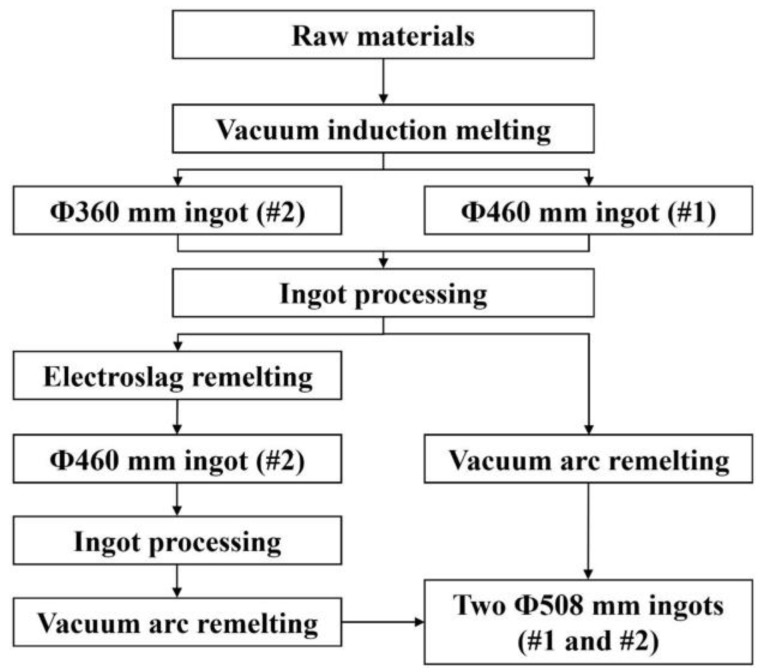
Schematic of the melting process.

The ESR-refined ingot #2 was turned to avoid surface pollution by the slag during subsequent melting and refining. Ingots #1 and #2 were both re-melted by VAR (ALD, Hanau, Germany). The melt rate was maintained at 7–9 kg/min to form the molten pool and was steadily and gradually lowered at a constant rate of 4–5 kg/min during the steady melting stage. Towards the end of the process, the voltage and current were adjusted to gradually reduce the melt rate and provide a controlled hot top. A vacuum of less than 1 Pa was used during the entire refining process. Two Ф 508-mm alloy ingots were produced. The melting process is shown in Figure 1. 

### 2.2. Preparation and Testing

The contents of Al, Ti, C, N, S, and O elements in ingots #1 and #2 during the intermediate stages of melting under various process conditions were determined by elemental analysis using an oxygen/nitrogen/hydrogen analyzer (Horiba, Ltd., Kyoto, Japan) and a carbon/sulfur analyzer (Wuxi Jiebo Electrical Technology Co., Ltd., Wuxi, China), and by inductively coupled plasma optical emission spectroscopy (ICP-OES) (PerkinElmer, Inc., Waltham, MA, USA). The contents of these elements at various stages of melting were recorded and analyzed.

**Figure 2:**
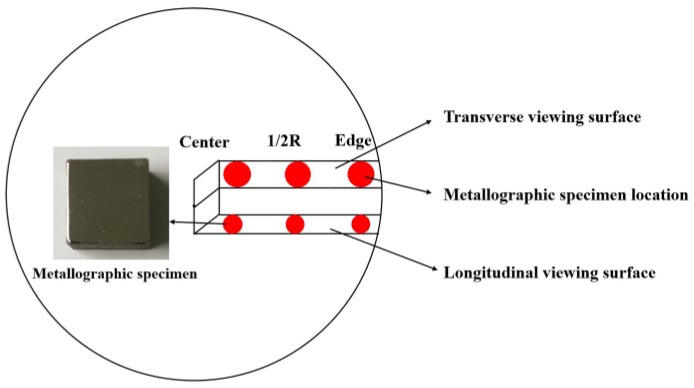
Schematic diagram of the metallographic specimen obtained.

Ten tensile samples of dimensions Φ 12 mm × 66 mm and five tensile samples of dimensions Ф 12 mm × 110 mm were extracted from the two Ф 508-mm alloy bars, respectively. Metallographic specimens of dimensions 15 mm × 15 mm × 15 mm were extracted from the centers, 1/2-R positions (R = radius), and edges of the longitudinal and transverse sections of the two Ф 508-mm alloy bars (Figure 2). The mechanical properties and inclusion phases of samples were determined by using a high-temperature cupping machine, a high-temperature fatigue testing machine, and an X-ray diffractometer (XRD) (Bruker Corporation, Karlsruhe, Germany).

The sizes, morphologies, and compositions of the inclusions were characterized by scanning electron microscopy with energy dispersive spectroscopy (SEM-EDS) (SEM, Phenom proX scanning electron microscopy, Eindhoven, The Netherlands). To obtain accurate inclusion data, each metallographic specimen was divided into four zones, with 25 different fields of view selected for the analysis of each inclusion and for recording purposes. The Image-Pro Plus software (Version 6.0, Media Cybernetics, Inc., Rockville, MD, USA) was used to analyze the average size and number of inclusions.

## 3. Results and Discussions

### 3.1. Chemical-Composition Analysis

The percentage contents of Al, Ti, C, N, S, and O in ingots #1 and #2 at various stages of melting and refining were statistically analyzed by the oxygen/nitrogen/hydrogen analyzer, the carbon/sulfur analyzer, and ICP-OES, and are listed in Table 2. The results reveal that the chemical compositions of the superalloys produced by the two different smelting processes meet technical requirements, but their elemental compositions and evolutions differ markedly. During VAR, the N content in ingot #1 dropped from 0.0067 wt.% to 0.0057 wt.%, i.e., a decrease of 14.9%, while the O content dropped from 0.0017 wt.% to 0.0012 wt.%, a 29.4% decrease. After protective gas ESR, the burning loss of Al in ingot #2 was 5.5%, and the contents of S and O decreased significantly, by 66.7% and 52.9%, respectively. At this stage, the Al, S, and O contents of ingot #2 were much lower than those of ingot #1. After VAR, the composition of N in ingot #2 was 0.0056 wt.%. No significant changes were observed in the compositions of other elements.

Hence, VIM maintained lower levels of O, N, and S in the ingot, while VAR further reduced the amounts of O and N. However, since ingot #2 also underwent ESR, the percentage compositions of Al, S, and O in ingots #2 and #1 were markedly different, ascribable to S in the liquid metal migrating into the overlying liquid-state molten slag (CaO and CaF_2_) [21,22] at the slag/metal interface during ESR, which lowers the amount of S in the ingot. This process occurs via chemical reactions Equations (1) and (2), as shown below. Reaction between the [O] in the molten slag and the Al in the ingot occurs readily, resulting in the burning loss of aluminum in the ingot. Since ESR takes place in an argon-protected environment, a rise in oxygen content caused by high-temperature oxidation during electrode remelting was effectively avoided. The metal and slag were also in contact for longer because the melt rate was low, which prevents oxide inclusions in the ingot from increasing, thereby lowering the oxygen content of the ingot [23].

(1) [S]+(CaO)=(CaS)+[O]

(2) [Ca]+[S]=(CaS)

### 3.2. XRD Analyses

**Figure 3:**
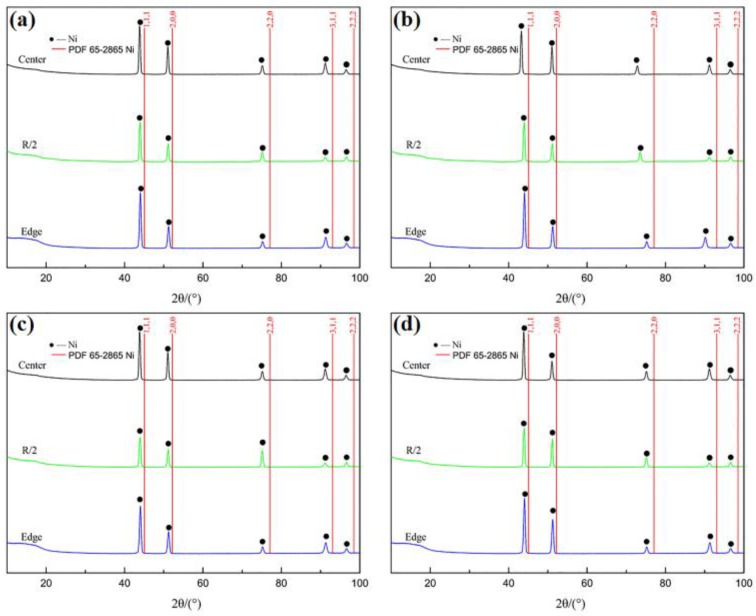
XRD patterns of GH4738 superalloy samples: (**a**) Ingot #1, transverse; (**b**) Ingot #1, longitudinal; (**c**) Ingot #2, transverse; and (**d**) Ingot #2, longitudinal.

The XRD spectra of samples collected at different positions of the ingots formed by the two different melting processes exhibited almost identical spectral features (Figure 3); hence, the spectra of an ingot prepared by any one of the melting and refining methods were analyzed in detail.

The XRD patterns exhibited no diffraction peaks attributed to inclusions at any sampling position. Only Ni diffraction peaks were observed, and they were shifted to smaller angles compared with those of the standard (PDF 65-2865 Ni), indicating that the Ni lattice had changed. Since most elements (Al, Ti, Mo, etc.) form solid solutions in superalloy matrices, the Ni-lattice spacings and lattice constants increase, shifting the Ni peaks to values lower than those of the standard [24]. Nevertheless, the remaining elements in the matrix were not part of the solid solution, and other harmful trace elements (S, N, etc.) in the superalloy formed inclusions; however, the levels in these inclusions were below the XRD detection limit and were not detected.

### 3.3. Inclusion Characteristics and Their Formation Mechanism

**Figure 4:**
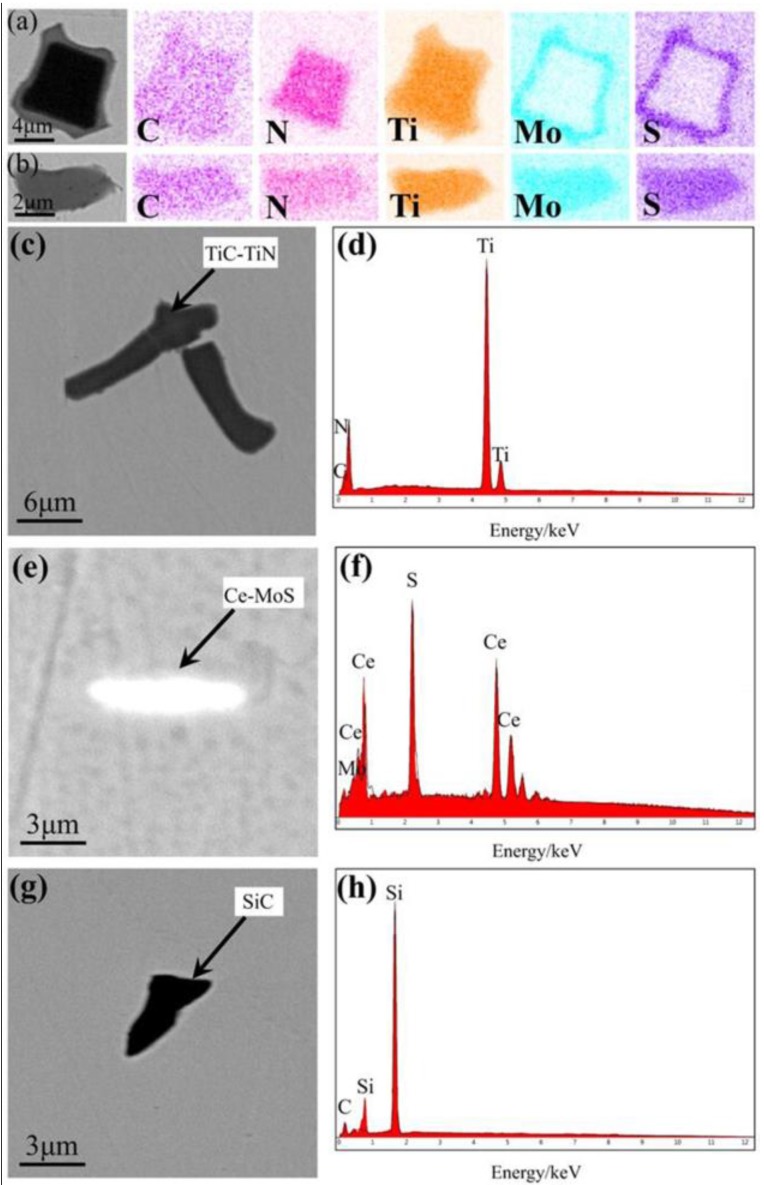
SEM images and energy dispersive spectroscopy (EDS) maps of the primary inclusions in ingot #1: (**a**,**b**) Ti(C, N)-MoS; (**c**,**d**) Ti(C, N); (**e**,**f**) Ce-MoS; and (**g**,**h**) SiC.

**Figure 5:**
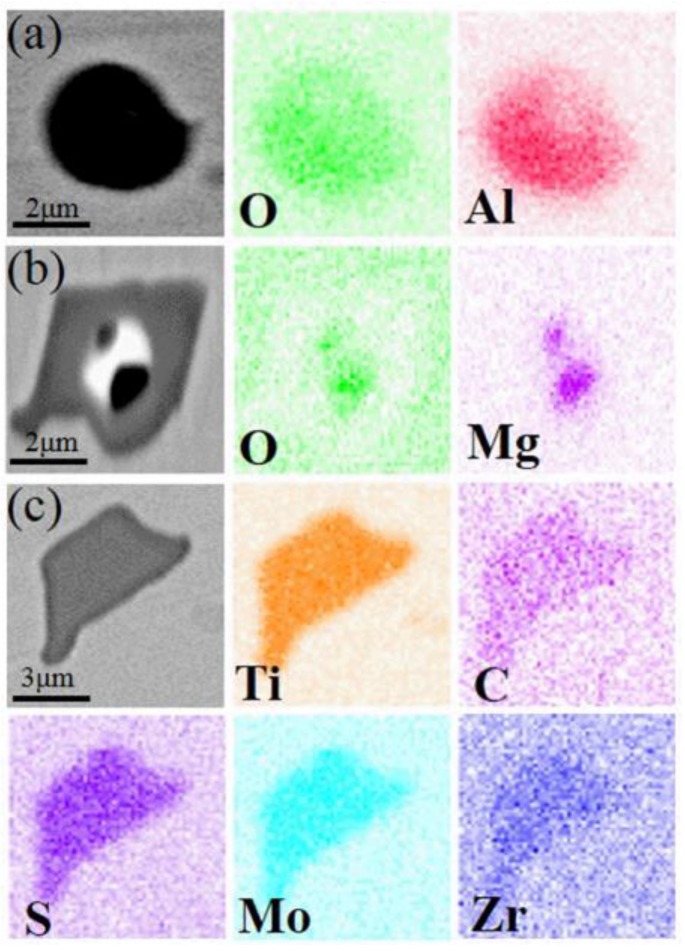
SEM images and EDS maps of other inclusions in ingot #1: (**a**) Al_2_O_3_; (**b**) MgO; and (**c**) Ti-Mo-Zr-C-S.

**Figure 6:**
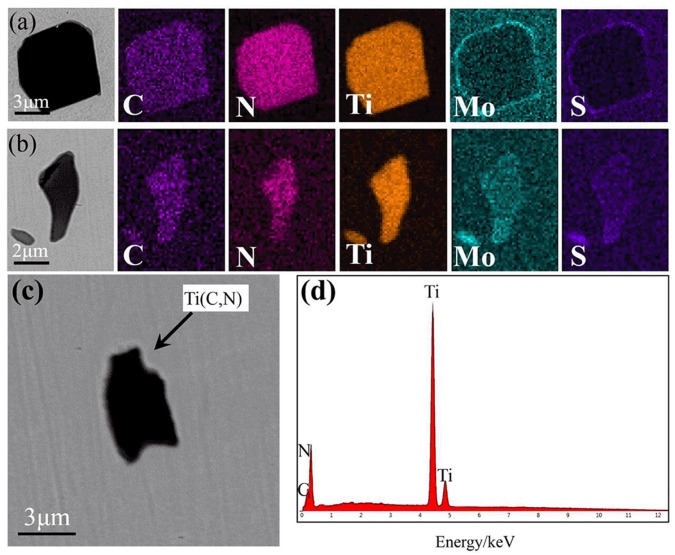
SEM images and EDS maps of the primary inclusions in ingot #2: (**a**,**b**) Ti(C, N)-MoS; and (**c**,**d**) Ti(C, N).

**Figure 7:**
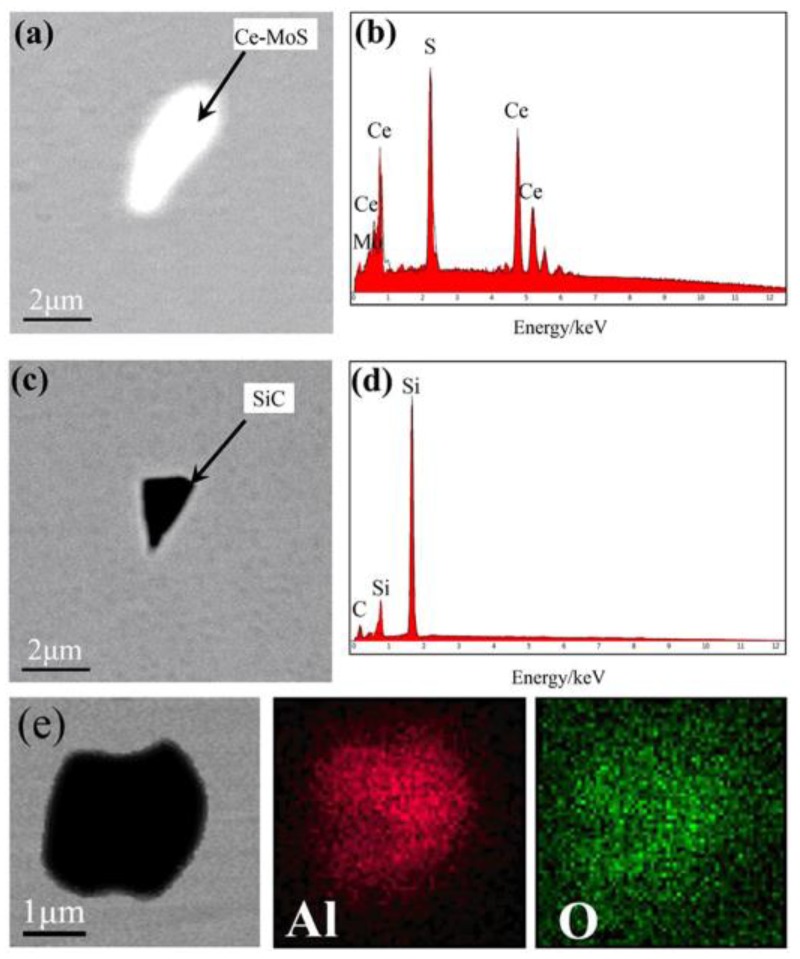
SEM images and EDS maps of other inclusions in ingot #2: (**a**,**b**) Ce-MoS; (**c**,**d**) SiC; and (**e**) Al_2_O_3_.

Analyses of the types, amounts, and sizes of the inclusions in all the superalloy samples can determine the influence of the smelting process. Inclusions at different sampling positions only exhibit minor variations in amount and size when the same smelting method was used. As such, only the types, amounts, and sizes of the inclusions formed using different melting modes were discussed in detail, and the relevant results are shown in Table 3 and Figure 4, Figure 5, Figure 6 and Figure 7.

As shown in Table 3, five types of inclusion were identified in the samples of superalloy (#1) refined by double melting. Four of these predominated: Ti (C, N)-MoS composite inclusions, Ti (C, N) composite inclusions, Ce-MoS inclusions, and SiC inclusions. Three types of inclusion were found in the triple-melted superalloy (#2) samples, with two of them predominating, viz. Ti (C, N)-MoS composite inclusions and Ti (C, N) composite inclusions. There were 5763 inclusions in the #1 samples, 1.68 times the number of inclusions in the #2 samples. The average inclusion size of the #1 samples was 0.6 μm larger than the #2 samples.

Figure 4a,b and Figure 6a,b illustrate the formation of two different forms of the Ti (C, N)-MoS composite inclusion in both ingots. In one of these, Mo and S elements were evenly distributed outside the inclusions, while in the other, they were evenly distributed in their interiors. This is explained by the melting of the superalloy, whereby the various types of inclusions collide, fuse, and become enriched at the surface owing to circulation and convection in the liquid metal and their densities, leading to the formation of composite inclusions [25]. In ingot #2, the even Mo and S distribution zones outside of the composite inclusions were noticeably less thick than those in ingot #1, owing to the low S content in #2, following ESR and its lack of precipitation alongside the inclusions, which reduces the thickness of the distribution.

Figure 4c,d and Figure 6c,d show the formation of Ti (C, N) composite inclusions in both ingots. This type of composite inclusion is composed of TiC and TiN. Since TiN has a lower standard Gibbs free energy of formation compared with TiC, it nucleates preferentially. TiC then precipitates on the TiN surface and grows to form TiC-TiN composite inclusions. Both TiN and TiC possess surface-centered cubic structures that differ little in their lattice constants. They dissolve into each other easily and form solid solutions during growth; consequently, they do not exhibit distinct nucleation centers [26].

Figure 4e–h and Figure 7a–d show that both ingots contained Ce-MoS and SiC inclusions, but in differing amounts. While dominant in ingot #1, these two inclusions were present in lower amounts in #2, which is ascribable to ESR effectively reducing the sizes and quantities of non-metallic inclusions, in addition to eliminating harmful elements (O, S, etc.) from the ingot. Therefore, the contents of Ce-MoS and SiC inclusions were lower in ingot #2. 

Further analyses of the Ce-MoS and SiC inclusions revealed that, although Ce was not present in the original GH4738 superalloy, it was introduced during the VIM process by the addition of revert GH4738 or impure raw materials to give Ce-MoS inclusions. SiC inclusions, on the other hand, were formed by the addition of C during VIM into the furnace as a deoxidizer for the removal of oxygen from the alloy solution through the formation of carbon monoxide in a carbon-oxygen reaction [27]. Since the oxygen concentration in the liquid metal decreases gradually, less CO was generated. Some CO became attached to the crucible wall because its bubbles were too small to be released. Hence, this gas reacts with elemental Si in the crucible wall to produce SiC inclusions [28]. 

Other types of inclusions were also present in small amounts in the ingots refined by either smelting method, as shown in Figure 5 and Figure 7. This indicates that inclusions formed by the segregation of some trace elements were unavoidable, although the double- and triple-melting processes maintained the contents of undesirable elements in the superalloy at low levels.

### 3.4. Analysis of Mechanical Properties 

**Figure 8:**
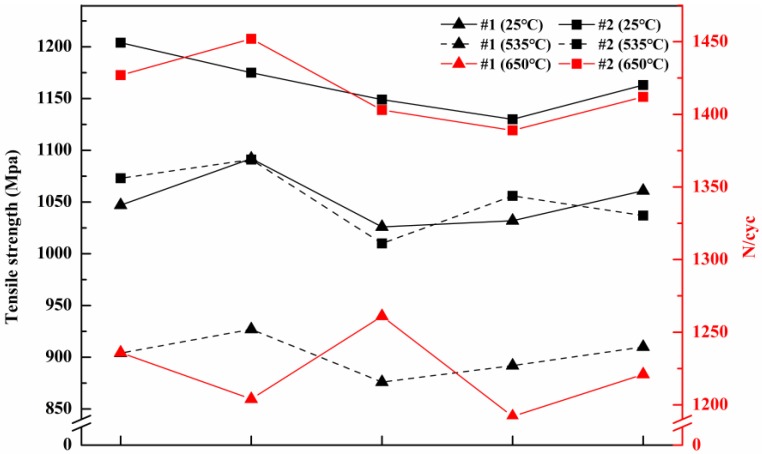
The mechanical properties of superalloy samples following different melting technologies.

The tensile samples of the ingots, formed by the different melting processes, were separately subjected to tensile tests at room temperature (25 °C) and high temperature (535 °C), and fatigue life test at high temperature (650 °C). The relevant results (Figure 8) reveal that the average tensile strength of the #2 tensile samples were 1164 MPa and 1053 MPa, respectively, when the tensile tests were performed at 25 °C and 535 °C, these values being higher than those of the #1 tensile samples by 113 MPa and 151 MPa, respectively. The average cycle number of #2 tensile sample was 1417, 1.16 times that of the #1 tensile sample, when the fatigue life was tested at 650 °C. Thus, the mechanical properties of the ingot #2 samples were better than those of the ingot #1 samples.

The amounts and sizes of the inclusions greatly influence the performance of the superalloy. Since the inclusions had different properties compared to the matrix, it would hinder the dislocation motion in the matrix during deformation, causing dislocation pile-up in front of the inclusions, resulting in local stress concentration [29]. As deformation was promoted, the inclusion broke or detached from the interface of the matrix when the concentrated stress reached the strength of the inclusion or the interfacial bonding strength between the inclusion and the matrix, resulting in microstructure defect and reduced properties of the superalloy [30]. Hence, improving the superalloy properties was based on the purity of superalloy.

### 3.5. Analysis of White-Spot Formation Probability

The above analyses show that, in terms of controlling the presence of harmful elements, the triple melting process maintained the O and S contents in ingot #2 at low levels. Ingot #2 contained fewer types and smaller amounts of inclusions than ingot #1. In terms of mechanical properties, the tensile strength and fatigue life of the ingot #2 samples were higher than those of the ingot #1 samples. Hence, triple melting provides a superalloy of higher purity and mechanical properties than double melting. However, the fact that white spots were also formed in superalloys refined by different melting techniques [31], which tarnish their purities, was often neglected. Although non-destructive testing did not locate any white spots in the two ingots smelted in this study, there was generally no guarantee that such defects would not form in the future. Therefore, in this section, we speculate on the factors that affect white-spot formation using the two melting techniques. 

The formation of white spots is closely related to the characteristics of the VAR procedure. In VAR, uneven heating of the electrode (ingot) may cause it to unexpectedly fall into the molten metal pool. Since the electrode has high density, it sinks to the bottom of the molten pool without fully melting and remains in the solidified ingot to form white spots [32]. Uneven heating of the electrode can occur because of electrode material pouring from above during VIM casting, which produces open and closed shrinkage defects in the electrode. Thin lattice texture, burrs, depressions, and nodular protrusions, among others, are found inside these shrinkage holes. Large internal stresses during the cooling of the electrode can also give rise to transverse cracks, further augmenting these defects [33]. Heating and heat conduction in the electrode is severely impeded at the loci of these defects, resulting in inhomogeneous heating of the electrode. ESR eliminates spongy shrinkage holes in the VIM-cast ingot to provide a more compact texture, which effectively improves the quality of the VAR metal electrode; the risk of white-spot formation in the superalloy is thereby minimized [34]. On the basis of the above discussion, we conclude that triple melting provides superalloys with high purities and stabilities.

## 4. Conclusions

Superalloy purity has come to limit progress in the superalloy field. The influence of the different melting techniques on superalloy purity had thus far been unclear. It is difficult to precisely determine the melting technique best suited for the preparation of a high-purity superalloy, which limits further improvements in superalloy performance. Strict control of superalloy purity is of particular importance. Therefore, herein we analyzed the effects of two melting techniques, viz. double melting, and triple melting, on chemical composition (various stages of melting and refining), inclusion characteristics and their formation mechanism, mechanical properties and white-spot formation probability in GH4738 superalloy. This study could serve as a guide for the selection of appropriate melting techniques for improving superalloy purity; in this sense, it also provides a theoretical foundation. The following conclusions were drawn from this study:

1. Double melting reduced the levels of N, S, and O in the GH4738 superalloy to 0.0057 wt.%, 0.002 wt.%, and 0.0012 wt.%, respectively, while triple melting reduced the contents of these elements to 0.0056 wt.%, 0.0007 wt.%, and 0.0008 wt.%, respectively. Hence, triple melting maintains lower levels of these harmful elements in the superalloy.
2. The superalloy samples refined by both techniques exhibited only Ni diffraction peaks in their XRD spectra, which were shifted to lower values compared with the standard peak positions (PDF 65-2865 Ni).
3. Four primary types of inclusion were present in the double-melted GH4738 superalloy samples, the number of inclusions was 5763 and the average inclusion size was 3.1 μm. Two dominant types of inclusion were present in the triple-melted superalloy samples; the number of inclusions was 3412 and the average inclusion size was 2.5 μm. Hence, triple melting generates fewer types and amounts of inclusion and the average inclusion size is also smaller.
4. The average tensile strengths of the double-melted GH4738 superalloy samples were 1051 and 902 MPa at 25 °C and 535 °C, respectively, and its average cycle number was 1223. The average tensile strengths of the triple-melted GH4738 superalloy samples were 1164 and 1053 MPa at 25 °C and 535 °C, respectively, and its average cycle number was 1417. Hence, triple melting produces higher mechanical properties in the superalloy.

## Figures and Tables

**Table 1:** Chemical composition of the GH4738 superalloy (wt.%).

Cr	C	Co	Mo	Al	Ti	S	Ni
18.0–21.0	0.03–0.1	12.0–15.0	3.5–5.0	1.2–1.6	2.75–3.25	<0.015	Balance

**Table 2:** Percentage compositions of some elements in superalloy ingots following different smelting processes (wt.%).

Melting Processes	Al	Ti	C	N	S	O
VIM (#1)	1.47	2.97	0.07	0.0067	0.0021	0.0017
VAR (#1)	1.45	2.96	0.07	0.0057	0.0020	0.0012
VIM (#2)	1.46	2.96	0.071	0.0066	0.0021	0.0017
ESR (#2)	1.38	2.94	0.071	0.0065	0.0007	0.0008
VAR (#2)	1.37	2.94	0.07	0.0056	0.0007	0.0008
Standard requirement	1.2–1.6	2.75–3.25	0.03–0.1	≤0.01	≤0.015	≤0.005

**Table 3:** Types, amounts, and sizes of inclusions in superalloy samples following different melting technologies.

Inclusions	VIM + VAR(#1)	VIM + ESR + VAR(#2)
Ti(C, N)-MoS	4299	2873
Ti(C, N)	524	458
Ce-MoS	409	——
SiC	398	——
Others	133	81
Total Inclusions	5763	3412
Average Inclusion Size (μm)	3.1	2.5
